# Live neighbor-joining

**DOI:** 10.1186/s12859-018-2162-x

**Published:** 2018-05-16

**Authors:** Guilherme P. Telles, Graziela S. Araújo, Maria E. M. T. Walter, Marcelo M. Brigido, Nalvo F. Almeida

**Affiliations:** 1 Instituto de Computação, Universidade Estadual de Campinas, Cidade Universitária, Campinas, 13083-852 Brazil; 2 Faculdade de Computação, Universidade Federal de Mato Grosso do Sul, Av. Costa e Silva, s/n, Campo Grande, 79070-900 Brazil; 3 Departamento de Ciência da Computação, Universidade de Brasília, Campus Darcy Ribeiro, Brasília, 70910-900 Brazil; 4 Instituto de Ciências Biológicas, Universidade de Brasília, Campus Darcy Ribeiro, Brasília, 70910-900 Brazil

**Keywords:** Phylogeny, Live phylogeny, Neighbor-joining

## Abstract

**Background:**

In phylogenetic reconstruction the result is a tree where all taxa are leaves and internal nodes are hypothetical ancestors. In a live phylogeny, both ancestral and living taxa may coexist, leading to a tree where internal nodes may be living taxa. The well-known Neighbor-Joining heuristic is largely used for phylogenetic reconstruction.

**Results:**

We present Live Neighbor-Joining, a heuristic for building a live phylogeny. We have investigated Live Neighbor-Joining on datasets of viral genomes, a plausible scenario for its application, which allowed the construction of alternative hypothesis for the relationships among virus that embrace both ancestral and descending taxa. We also applied Live Neighbor-Joining on a set of bacterial genomes and to sets of images and texts. Non-biological data may be better explored visually when their relationship in terms of content similarity is represented by means of a phylogeny.

**Conclusion:**

Our experiments have shown interesting alternative phylogenetic hypothesis for RNA virus genomes, bacterial genomes and alternative relationships among images and texts, illustrating a wide range of scenarios where Live Neighbor-Joining may be used.

## Background

Neighbor-Joining [1] is a widely used heuristic for phylogenetic reconstruction from a distance matrix. It has been applied to many biological datasets and also to non-biological data, including text and image [2]. Neighbor-Joining is recognized by rapidly building phylogenies that are close to the optimal when the number of taxa is not too large.

In a live phylogeny [3] we admit that both ancestral and current taxa coexist. This is likely to happen for instance with viruses, that evolve at high rates [4–7]. As the reconstruction of traditional phylogenies, the reconstruction of live phylogenies is also computationally hard. We must then resort to heuristics for obtaining solutions that are as good as possible within reasonable amounts of time and computer resources.

In this article we introduce a heuristic named Live Neighbor-Joining to reconstruct live phylogenies, built on the same ground of Neighbor-Joining. We have applied Live Neighbor-Joining to different sets of viral and bacterial genomes, thus introducing different hypothesis for the relationship of those species. We also illustrate the usage of Live Neighbor-Joining on non-biological datasets.

### Neighbor-Joining

Suppose that *U* is the set of numeric taxonomic units under study, and suppose also that they are labeled {1,2,…,*n*}. If an *n*×*n* matrix *D* of real numbers representing distances among taxonomic units *U* is given, solving the phylogenetic reconstruction problem is to build an unrooted tree *T* whose internal nodes have degree 3, whose leaves are in one-to-one correspondence with taxa in *U*, and whose edges are labeled with real numbers such that the sum of edge labels in the path between leafs *i* and *j* is equal to *D*_*ij*_. Such tree *T* is a phylogeny for *U*.

The phylogenetic reconstruction problem is computationally hard, except when *D* is additive. Additivity does not occur often in practice because of experimental errors and because measuring distance among taxa is also difficult. When *D* is additive, a polynomial time algorithm exists to build a phylogeny [8]. When *D* is not additive, the problem of finding a tree that minimizes the deviation to *D* is NP-complete [9] and heuristics are used to solve the problem in practice.

Neighbor-Joining (NJ) was introduced by Saitou and Nei [1] based on the idea of minimizing the sum of branch lengths in the final topology. The input is an *n*×*n* matrix *D* with pairwise distances among taxa in *U*.

**Fig. 1:**
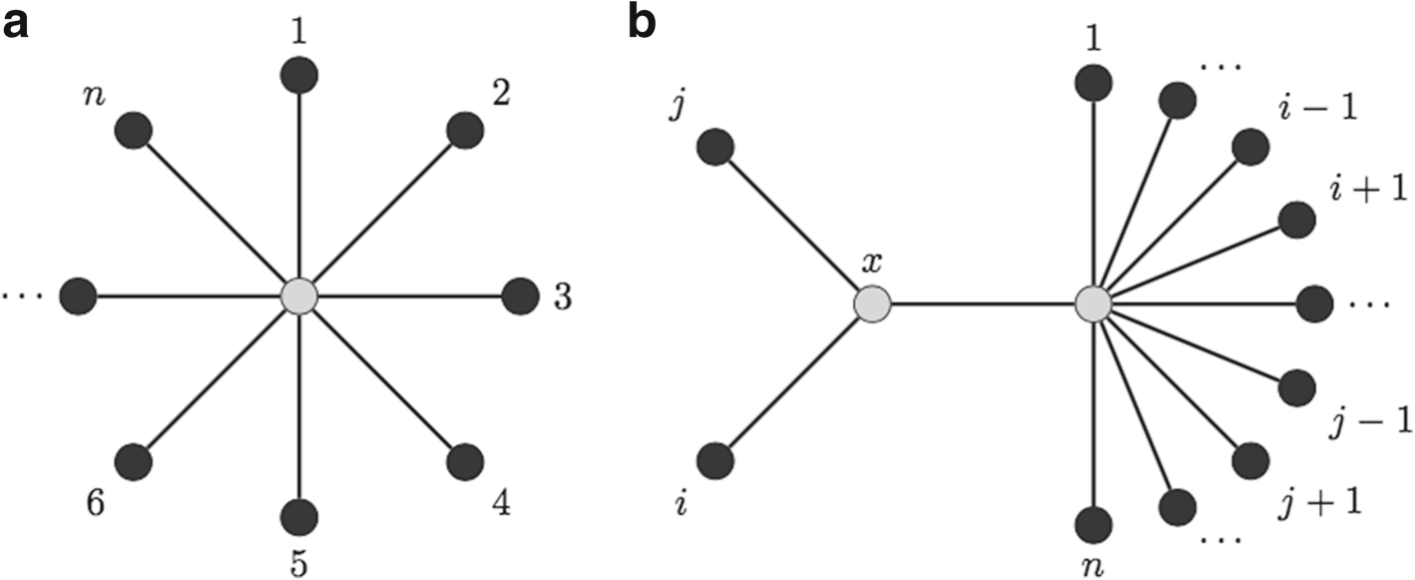
NJ sum of branch lengths. **a** A star with *n* taxa as leafs. **b** A hypothetical ancestor *x* is added between taxa *i* and *j*

If the *n* taxa in *U* form a star (Fig. 1a) and *i* and *j* are grouped as children of a hypothetical ancestor *x* (Fig. 1b), then the *S* score is defined from the sum of branch lengths:

1 $$ {\begin{aligned} S_{ij}&=\frac{1}{2(n-2)}\underset{\begin{array}{c} 1\leq k\leq n\\ k\not=i,j \end{array}}{\sum}(D_{ik}+D_{jk})\\&\quad+ \frac{D_{ij}}{2} + \frac{1}{n-2}\underset{\begin{array}{c} 1\leq k<\ell\leq n\\ k,\ell\not=i,j \end{array}}{\sum} D_{k\ell}. \end{aligned}}  $$

At each iteration, NJ evaluates *S* for each pair of taxa, selects the pair {*i*,*j*} with the least value and reduces *U* by removing *i* and *j* and adding taxon *x*. The distance between *x* and *y*∈*U*∖{*i*,*j*} is evaluated as

$$D_{xy}=\frac{D_{iy} + D_{jy} - D_{ij}}{2}. $$

Let the average distance between *i* and other taxa not including *j* be

$$D_{i\setminus j} = \frac{{\sum\nolimits}_{\begin{array}{c}1\leq k\leq n\\ k \not=j \end{array}}D_{ik}}{n-2}. $$

NJ then adds *x* as the ancestor of *i* and *j* as in Fig. 1b with branches *L*_*ix*_ and *L*_*jx*_ calculated as

2 $$\begin{array}{@{}rcl@{}} L_{ix}=\frac{D_{ij} + D_{i\setminus j} - D_{j\setminus i}}{2}, \quad L_{jx}=\frac{D_{ij} + D_{j\setminus i} - D_{i\setminus j}}{2}.  \end{array} $$

When only three taxa *i*, *j* and *k* are left, NJ joins them by a common ancestor *x*, sets branch lengths as below and terminates.

3 $$ \begin{aligned} L_{ix} &= \frac{D_{ij} + D_{ik} - D_{jk}}{2}, \;\; L_{jx} = \frac{D_{ij} + D_{jk} - D_{ik}}{2}, \\ L_{kx} &= \frac{D_{ik} + D_{jk} - D_{ij}}{2}  \end{aligned}  $$

NJ runs in *O*(*n*^3^) time. For dealing with large phylogenies, other heuristics based on NJ improve the running time, sometimes sacrificing precision, for instance [10–17].

### Live phylogeny

Solving the live phylogeny reconstruction problem [3] is to build an unrooted tree *T* whose internal nodes have degree 3, such that there is a subset *V* of the nodes of *T* that includes all leaves and is in one-to-one correspondence to *U*, and whose edges are labeled with real numbers such that the sum of edge labels in a path between nodes *i*,*j*∈*V* is equal to *D*_*ij*_. Such tree *T* is a live phylogeny for *U*.

Live phylogeny is also easy for the additive case and hard for the non-additive case [18], where it has been shown that, when an additive matrix is given NJ will build a tree with zero-length edges for a live phylogeny. The authors present a heuristic that combines a search for zero-length edges and a search for triples of internal nodes with a “non-congruent” distance relation and replaces a hypothetical node with a live internal node (following the approach introduced in [19]). Because there is no available benchmark for live phylogeny and a branch-and-bound is not known for the problem, the heuristic was evaluated against NJ on instances with different non-additivity scores, trying to resemble the problem difficulty with respect to additivity.

## Methods

**Fig. 2:**
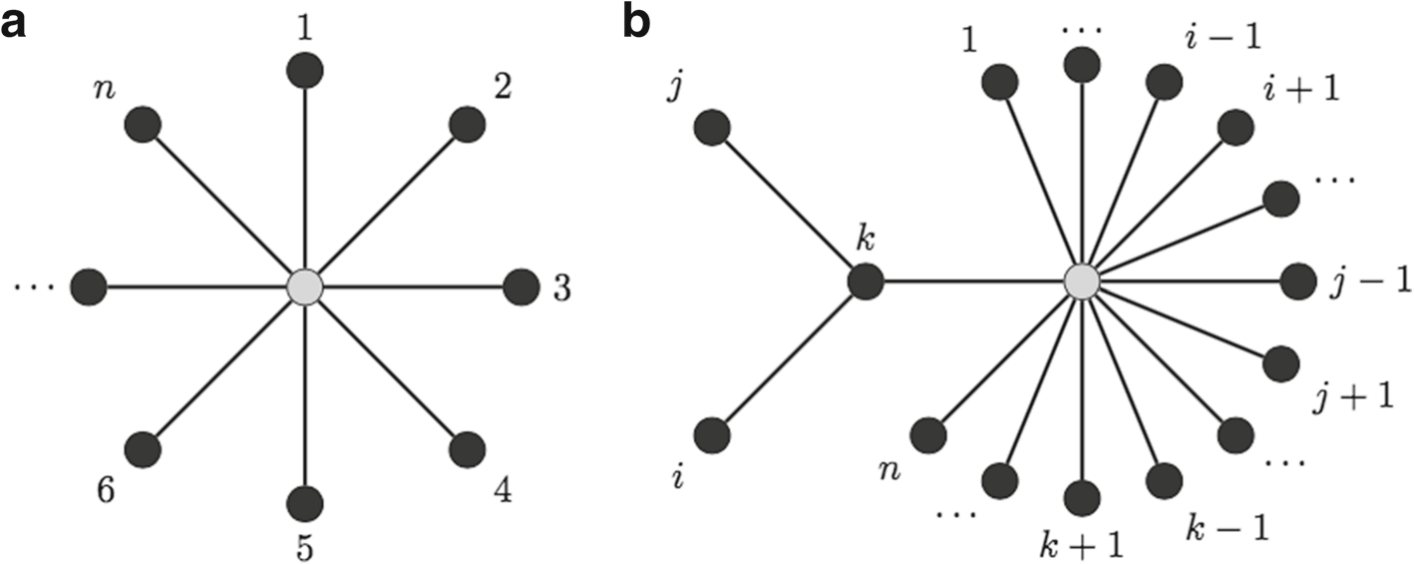
LNJ sum of branch lengths. **a** A star with *n* taxa as leafs. **b** A live ancestor *k* is added between taxa *i* and *j*

Live Neighbor-Joining (LNJ) extends the numeric rationale of Neighbor-Joining introducing the case where a live ancestor results in a smaller sum of branch lengths. If the *n* taxa form a star (Fig. 2a), and *i* and *j* are grouped as children of taxon *k* that is a leaf (Fig. 2b), then the sum of branch lengths will be

$$L_{ik} + L_{jk} + \underset{\begin{array}{c} 1\leq v\leq n\\ v\not=i,j,k \end{array} }{\sum}L_{vx}. $$

We define the *T* score as

4 $$\begin{array}{@{}rcl@{}} T_{ijk}=D_{ik} + D_{jk} + \frac{{\sum\nolimits}_{ \begin{array}{c} 1\leq u<v\leq n\\ u,v\not=i,j \end{array} }D_{uv}}{n-3}.  \end{array} $$

At each iteration, LNJ will select either the pair or the triple with least score. When a pair is selected, LNJ just proceeds like NJ does. When a triple is selected, *U* will be reduced by the removal of *i* and *j*, *k* will be the live ancestor of both *i* and *j* and branch lengths will be

$$L_{ik}=D_{ik}, \quad L_{jk}=D_{jk}. $$

At the end, when three nodes are left, they are connected through a hypothetical ancestor, as in NJ. If only two nodes are left, they are connected by an edge (*i*,*j*) whose weight is *D*_*ij*_.

The pseudo-code for LNJ is shown below. The input is the *n*×*n* distance matrix *D*, which is also regarded as *U*. If the sum of all distances in *D* and the sum of distances from each node to the others are kept by the algorithm, and if an array of flags is used to keep track of live ancestors, then evaluating *T* at each iteration takes *O*(*n*^3^). Updating *D* and the sums takes *O*(*n*). Then LNJ will run in *O*(*n*^4^) time and *O*(*n*) additional space.



We have evaluated the running time of our implementations on increasing input sizes. We generated 10 random additive matrices of each size and averaged the running time. LNJ was forced to always select a pair of nodes instead of a triple (tempering with Line 5 of the algorithm), ensuring that the number of nodes always decreases by one and that the number of matrix accesses is maximized. We can see in Table 1 that the running times are stable, growing by factors close to 8 for NJ and close to 16 for LNJ when the input size is doubled. The experiments were executed on a system with an Intel Xeon E5-2630-v3 processor at 2.40 GHz with 20MB cache, 384 GB of RAM and a 13 TB SATA storage, running a 64-bit GNU/Linux (Debian 8, kernel 3.16.7). The sources where compiled by GCC 4.9.2 with -O3. Table 1 also shows the peak memory usage, which is the same for NJ and LNJ.

**Table 1:** Running times

n	NJ time (s)	LNJ time (s)	Peak memory (B)
128	0.0032	0.0318	115,763
256	0.0254	0.4234	360,755
512	0.2020	6.1179	1,244,275
1024	1.6120	92.0992	4,584,227
2048	12.9061	1,425.4783	17,557,539
4096	103.3255	22,410.6324	68,669,987

Average time in seconds for Neighbor-Joining and Live Neighbor-Joining for different numbers of species, and peak memory usage in bytes

## Results

RNA virus consists in a very good environment for testing approaches for live phylogeny, since they present the highest mutation rates among living beings, evolving too fast and possibly coexisting [5–7]. Here we present the application of Live Neighbor-Joining to three different sets of RNA virus genomes: Zika, Chikungunya and Ebola.

The input for each set is a whole genome distance matrix built using the package MUMi [20], which generates what is called MUM genomic distance index for each pair of genomes based on criteria of diversity, like average nucleotide identity and proportions of DNA shared by both genomes. The MUM index is calculated after running MUMmer [21], a very popular tool for whole genome pairwise alignment based on suffix trees and seeds called MUMs (Maximal Unique Matches). MUMi values are always in the interval [ 0,1] and are inversely proportional to the number of MUMs found between both genomes. So, the higher MUMi is, the more distant are the genomes being compared [22].

All input distance matrices used in the datasets, the corresponding trees in Newick format [23], and also the source code of LNJ are available at https://git.facom.ufms.br/bioinfo/LNJ. The trees shown below were drawn using FigTree (http://tree.bio.ed.ac.uk/software/figtree), PhyD3 (https://phyd3.bits.vib.be), GraphViz dot (https://www.graphviz.org) and D3.js (https://d3js.org).

### Zika virus

Zika virus is an RNA virus of the family *Flaviviridae*, genus *Flavivirus*, and it is spread by *Aedes* mosquitoes, such as *A. aegypti* and *A. albopictus*. Outbreaks have been recently reported in Americas and Africa. Because Zika virus infection during pregnancy has been associated with birth defects, like microcephaly, it has attracted considerable attention of the scientific community. Lanciotti et al. [24], for instance, presented a phylogeny of 20 Zika virus strains, derived by Neighbor-Joining methods bootstraped 1,000 times. Here we propose an alternative topology to the same set of genome sequences.

**Fig. 3:**
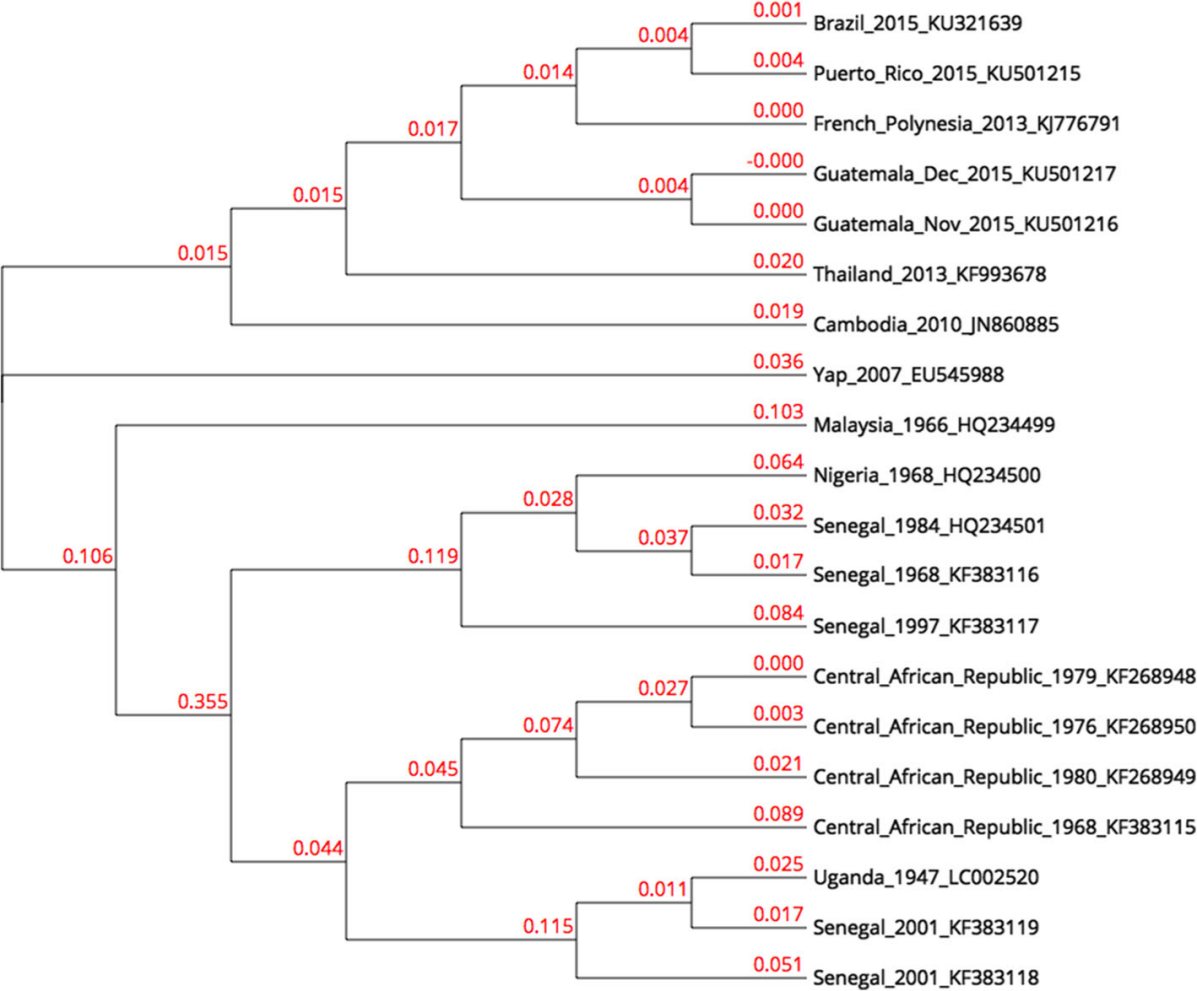
Zika NJ tree. NJ tree for 20 Zika virus genomes

**Fig. 4:**
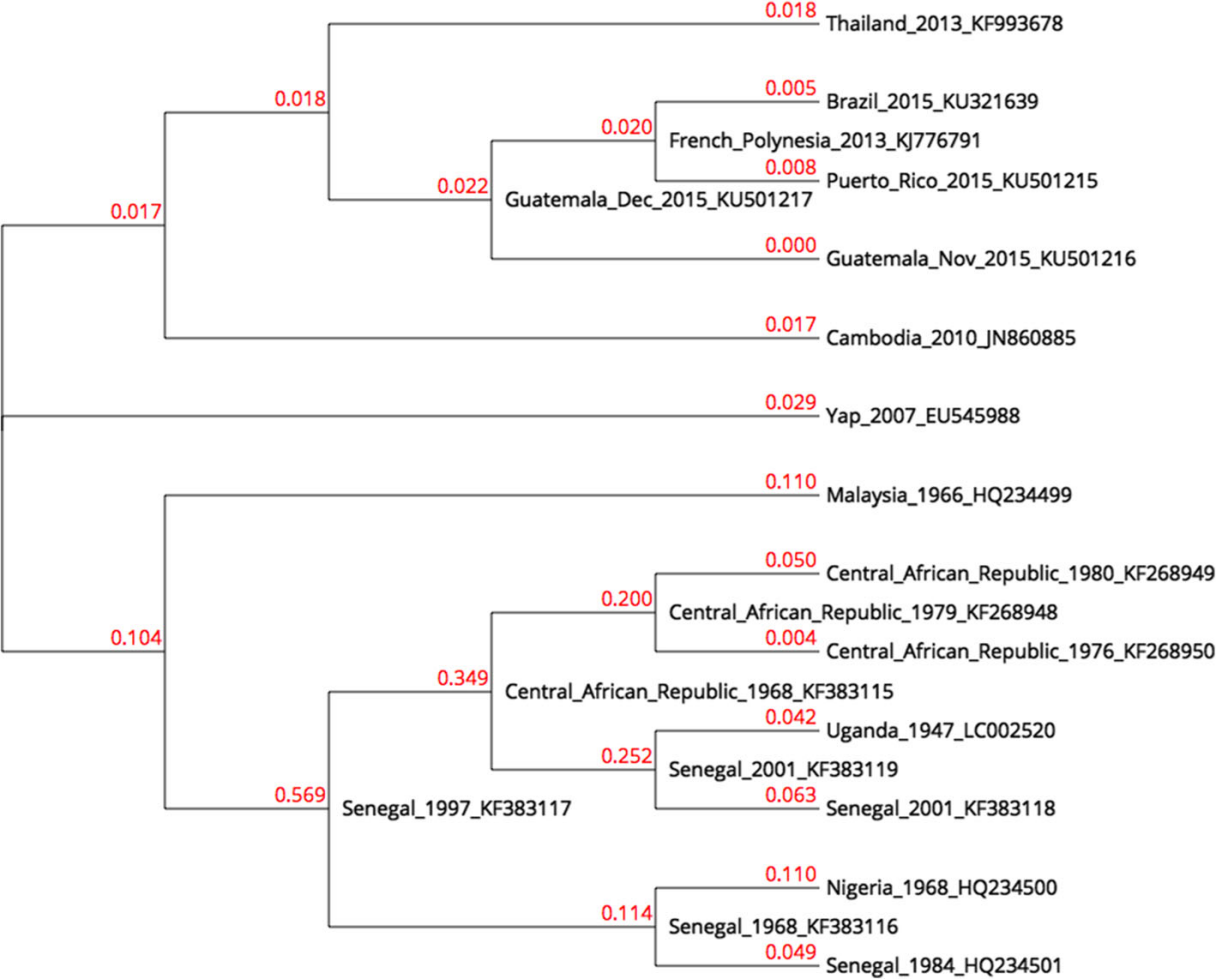
Zika LNJ tree. Live Neighbor-Joining tree for 20 Zika virus genomes

In order to build our live phylogeny, a distance matrix for the same 20 Zika virus genomes using the pipeline described above was built. Genome lengths range from 10,247 to 10,807 bases. We built a phylogeny for this matrix using NJ, shown in Fig. 3. The live phylogeny built by LNJ is shown in Fig. 4.

The three groups identified by Lanciotti et al. (East African, West African and Asian) are grouped in subtrees in the NJ tree, except for Yap 2007 that was positioned further from other members of the Asian group. The LNJ tree has East African as a distinctive subtree rooted by a member of the West African group, whose other members also form a distinctive subtree. In the LNJ tree, 7 virus become live ancestors, introducing hypothesis that could be considered in a deeper analysis of the alignments among these genomes.

The predicted live ancestors (Fig. 4) did not change the overall topology built by NJ (Fig. 3), but improved it, suggesting how virus populations are evolving. Zika virus was discovered in Africa in the 1950’s and all African isolates are grouped in both NJ and LNJ analyses, but the LNJ method suggested that KF383117 sequence corresponds to the precursor of today’s circulating virus. Interestingly, KF383117 sequence was registered in 1997, later than other isolates that date as back as 1968. Since LNJ poses KF383117 in the first node of an African sub-tree, it is reasonable to consider it as the closer sequence to the common ancestor of the African sequences analyzed in this work.

The 2015 Zika epidemic in South America is supposed to have arrived from the Polynesian athletes that landed in Brazil for a world Canoe championship [25]. Data in Fig. 4 supported that hypothesis since the French Polynesia sequence KJ776791 is placed as live ancestor of both a Brazilian and a Puerto Rican sequences. However, this French Polynesian sequence may have evolved from an earlier American sequence, as suggests the Guatemalan sequence KU501217, though the Polynesian Zika isolate may have arrived from America rather than Asia or Africa.

### Chikungunya virus

Another recently noticed important virus is Chikungunya, of family *Togaviridae*, genus *Alphavirus*. The main infection symptoms are fever and joint pain. The same female mosquitoes that transmit Zika virus spread Chikungunya virus. That is why this virus has also attracted attention from researchers.

Nunes et al. [26] investigated the origins and the potential for spreading of Chikungunya virus in Brazil from the first cases confirmed in 2014. According to them, four genotypes have been identified since 1952, named by the regions where they have been found: East-Central-South-African (ECSA), West African, Asian and Indian Ocean (IOL). Phylogenies based on full-length genome sequences were estimated using maximum likelihood in [26]. A total of 76 genomes representing all four viral genotypes were used: 11 West African, 12 ECSA, 17 IOL, and 30 Asian, besides 6 new Brazilian strains. Genome sizes range from 11,569 to 12,189. Nunes and colleagues concluded that the strains associated with the early-phase outbreaks in Brazil belong to the Asian and ECSA genotypes.

**Fig. 5:**
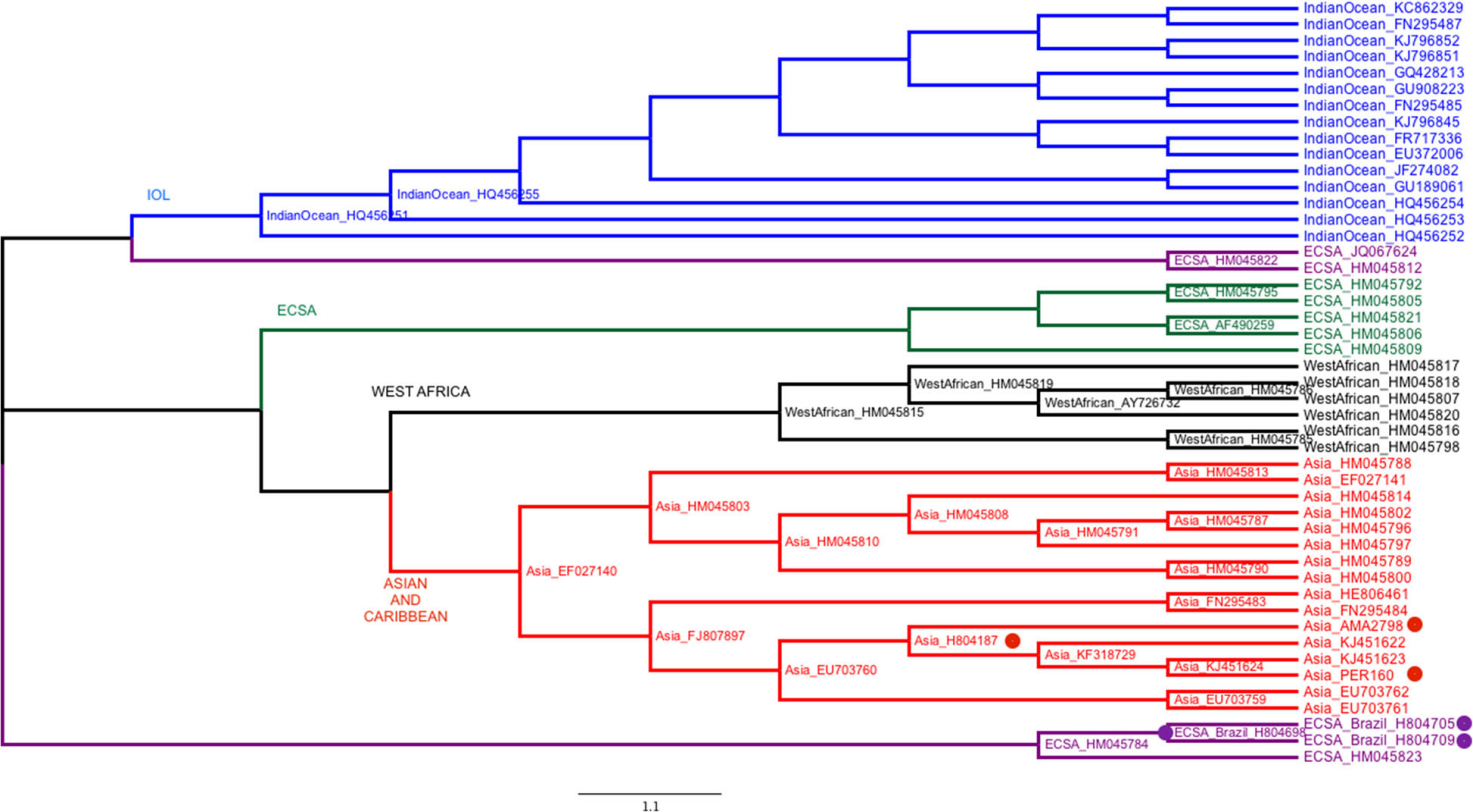
Chikungunya LNJ tree. Live Neighbor-Joining tree for 74 Chikungunya virus genomes. Dots represent Brazilian strains (purple strains are from Bahia State and red ones from Pernambuco and Pará States)

By using the LNJ heuristic, we built a live phylogeny using 74 out of the 76 sequences used by Nunes and colleagues, since two of them (CNR20235 and CNR20236) were not found in Genbank, after following their indications. The live phylogeny is shown in Fig. 5, with 27 live internal nodes.

**Fig. 6:**
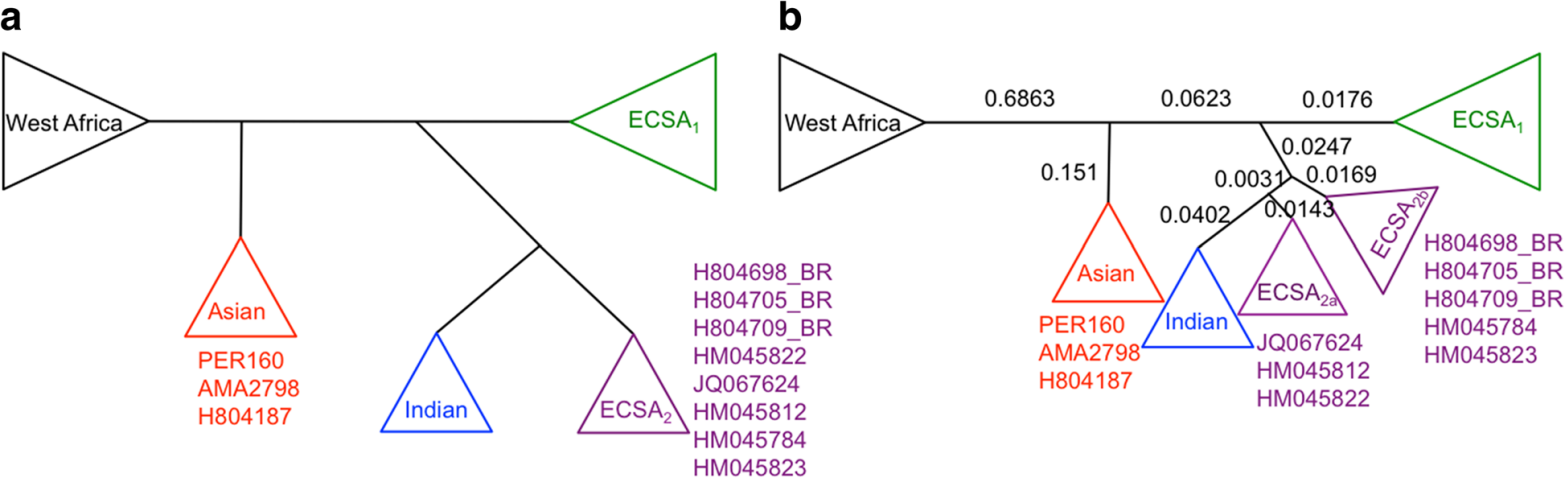
Chikungunya high level tree topologies. **a** Nunes et al. [26] and **b** LNJ high level topologies. Brazilian strains from Bahia (purple dots in Fig. 5) have been separated from the other ECSA_2_ ones (red dots) in our tree

Figure 6 shows a high level representation for the clustering of the genotypes in both Nunes et al. and our topologies. Although ECSA_2_ genotype in Nunes topology (Fig. 6a) became separated in two neighboring groups in our tree (Fig. 6b), the Brazilian strains H804698, H804705 and H804709 (from Feira de Santana, Bahia state) were clustered on a better way, with H804698 being ancestor of the other ones, as shown in Fig. 5. At the same time, the distances in LNJ topology show how close are ECSA _2*a*_ and ECSA _2*b*_.

Nunes et al. proposed that Chikungunya sequences from Brazilian isolates are derived from both Asian and ECSA genotypes. Among the Asian classified sequences, only one was considered an autochthonous case (AMA2798). Interestingly, LNJ analysis suggests that it is derived from H804187, a sequence isolated from a patient that had traveled from the Caribbean island Guadalupe to the city of Belém in Brazil, though LNJ data suggests that patient P37 (AMA2798) infection has been derived from the virus imported by patient P34 (H804187). Moreover, the PER160 sequence (P25) should not be related to the virus circulating in Belém, since it seems to be derived from the KJ45164, a sequence isolated in the Caribbean Virgin Island, a fact in agreement with epidemiological data described in [26], that shows the patient P25 that had traveled to Dominican Republic.

The use of LNJ may help improve epidemiological investigation, suggesting a more accurate chain of infection of a virus outbreak. In the Feira de Santana autochthonous cases [26], LNJ analysis suggests that the virus infecting patients P38 (H804709) and P39 (H804705) had a common ancestor, namely H804698, that infected patient P36, though the latter patient may have been infected by a parental virus that further infected the other two patients, all of them living in the same geographical area. Therefore LNJ data could be exploited for understanding how a certain virus evolves along a chain of infection.

### Ebola virus

Unlike Zika and Chikungunya, Ebola virus, the causative agent of Ebola Disease (previously known as Ebola Hemorrhagic Fever), is transmitted among humans by direct physical contact with infected bodily fluids, mainly blood, faeces and vomit. The first known outbreak occurred in Zaire in 1976. The last reported one was in the Democratic Republic of the Congo, on May 2017 [27].

Dudas et al. [28] proposed a maximum likelihood tree bootstraped 100 times of 49 strains of Ebola virus. They used several genome sequences from Genbank (including Bundibugyo BDBV, Reston RESTV, Sudan SUDV, Tai Forest TAFV and Zaire EBOV strains) and the sequences from the recent Guinea 2014 outbreak. The genomic sequence lengths range from 18,774 to 18,961.

**Fig. 7:**
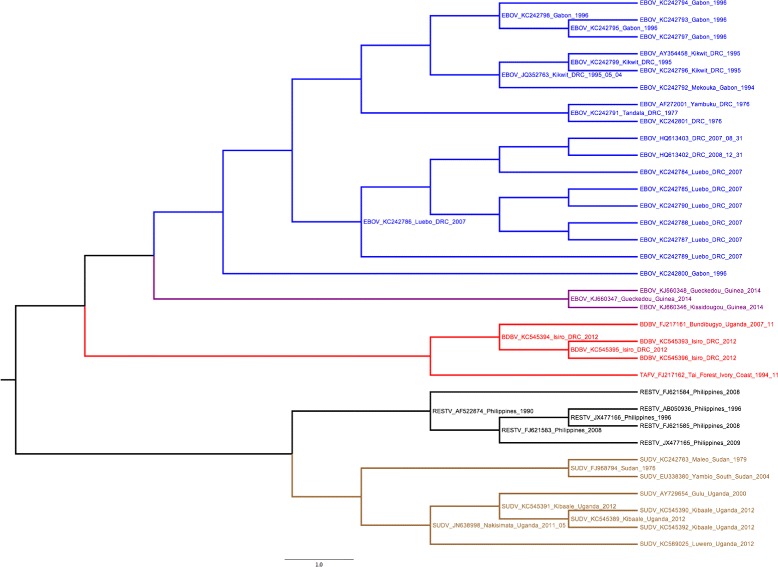
Ebola LNJ tree. Live Neighbor-Joining tree for the 49 Ebola virus sequences used in [28]

Using the same sequences, LNJ generated the phylogeny shown in Fig. 7, with all clades presented in [28] maintained, except for the exchange of strain KC242800 and the clade from Luebo. Besides, a strain from Gabon (1994) was positioned in the Kikwit clade. The BDBV clade was maintained with 2 live internal nodes, the RESTV clade with 3 live internal nodes and the EBOV clade with 7 live internal nodes.

The Ebola Virus strain Reston is the only known non-African species of Ebola, and is grouped as a unique clade by LNJ. The sub-tree marked in black in Fig. 7 is essentially the tree observed by Carroll et al. [29], obtained using Bayesian coalescent analysis. The differences are the prediction of sequences JX477166, FJ621583 and AF5222874 as internal nodes. Furthermore, LNJ predicts that FJ621584, while still an outgroup for Reston virus, evolves from AF5222874, that appear as the most internal node of this subtree, though LNJ seems to aggregate a temporal dimension in biological phylogeny.

## Discussion

Although live phylogeny looks more appropriate for very fast evolving organisms, like viruses, Live Phylogeny can also be used on other kind of organisms. Here we present a case study using LNJ on the following eight phylogenetic spread bacteria species (with their RefSeq assembly accessions and shortnames): *Azotobacter vinelandii* CA (GCF_000380335, Azoto), *Pseudomonas syringae* pv. *cerasicola* (GCF_900235885, Pseudo), *Escherichia coli* str. K-12 (GCF_000005845, Ecoli), *Xylella fastidiosa* str. DSM 10026 (GCF_900129695, Xylella), *Xanthomonas fuscans* subsp. *fuscans* 4834-R (GCF_000969685, Xanthofuscans), *Xanthomonas axonopodis* pv. *citri* str. 306 (GCF_000007165, Xantho306), *Mycobacterium tuberculosis* H37Rv (GCF_000195955, Mtuberc) and *Mycobacterium bovis* AF2122/97 (GCF_000195835, Mbovis). These organisms clearly form four distinct clades, according to their hosts and respective causative diseases.

**Fig. 8:**
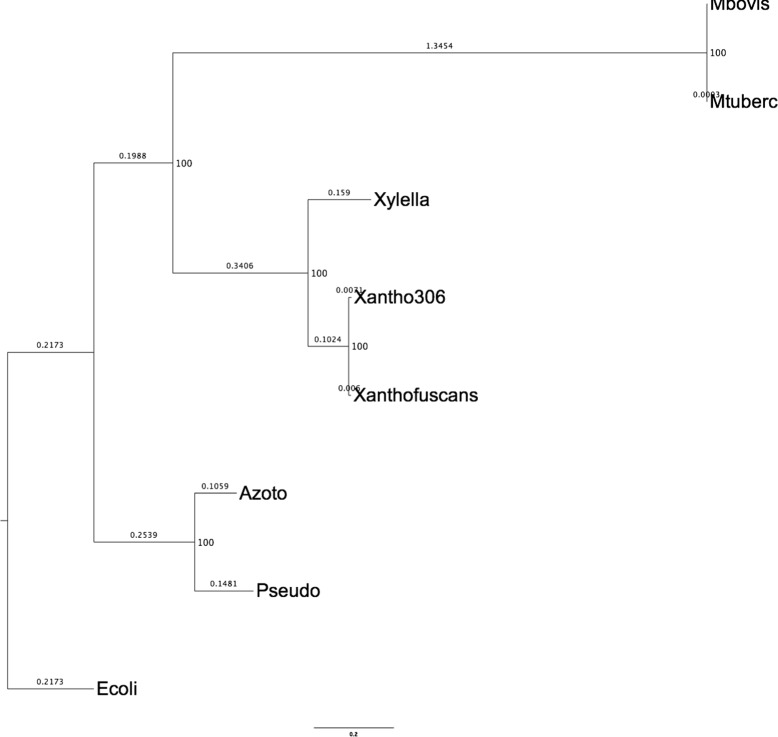
Orthologsorter tree. The numbers at nodes represent the bootstrap values

Figure 8 shows the topology obtained by Orthologsorter [22], an automatic pipeline to compare genomes in terms of their protein-coding gene content, using a supermatrix approach. Shortly, a whole multiple sequence alignment representing the concatenation of ortholog families is used as input to RAxML [30], which builds an unrooted phylogenetic tree, using by default the PROTCATJTT substitution model, with rapid bootstrapping (100 replicates) and subsequent Maximum Likelihood search.

**Fig. 9:**
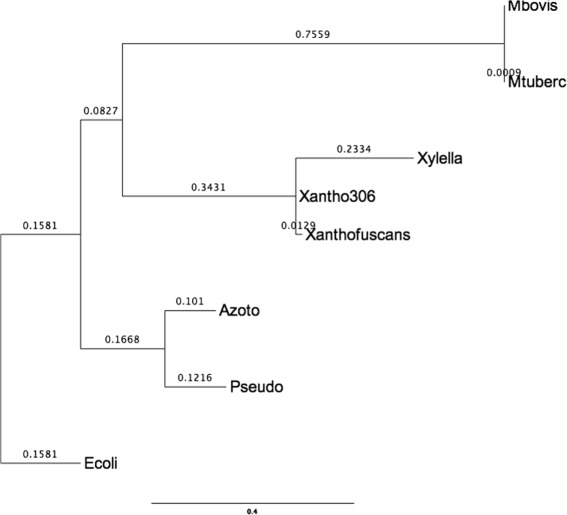
LNJ tree on the set of eight bacteria species. Xantho306 became a live internal node

Taking the same multiple sequence alignment, in this case containing 75,738 columns, we used PROTDIST [31] to build a distance matrix. Using this matrix, NJ has built the same topology as the one shown in Fig. 8. Figure 9 shows LNJ tree, which kept the same clades, but making Xantho306 a live ancestor of Xylella and Xanthofus.

As another approach using the same dataset, but at this time taking as input the whole chromosome sequence of each organism, we again used MUMi [20] to build an input distance matrix. Both NJ and and LNJ obtained the same topology as the one shown in Fig 8, with no internal live nodes. This can be explained by the fact that MUMi is based on whole DNA content, which includes large portions of transpositions (very common in bacteria) and could not capture similarities present in some shared proteins families.

### LNJ on non-biological data

In exploratory data visualization an important task is the construction of visual representations that enable users in the quest for groups of related data, in the discovery of relations among data items, in the identification of outliers and in other tasks [32]. Interaction and summarization tools are typically provided over such visual representations.

A widespread visual representation is built by mapping each data item onto a point in the visual space such that the more related their contents are, the closer their points are on the layout. This is a hard problem in general, and it has been solved in practice using dimensional reduction techniques, specially multidimensional projections [33].

**Fig. 10:**
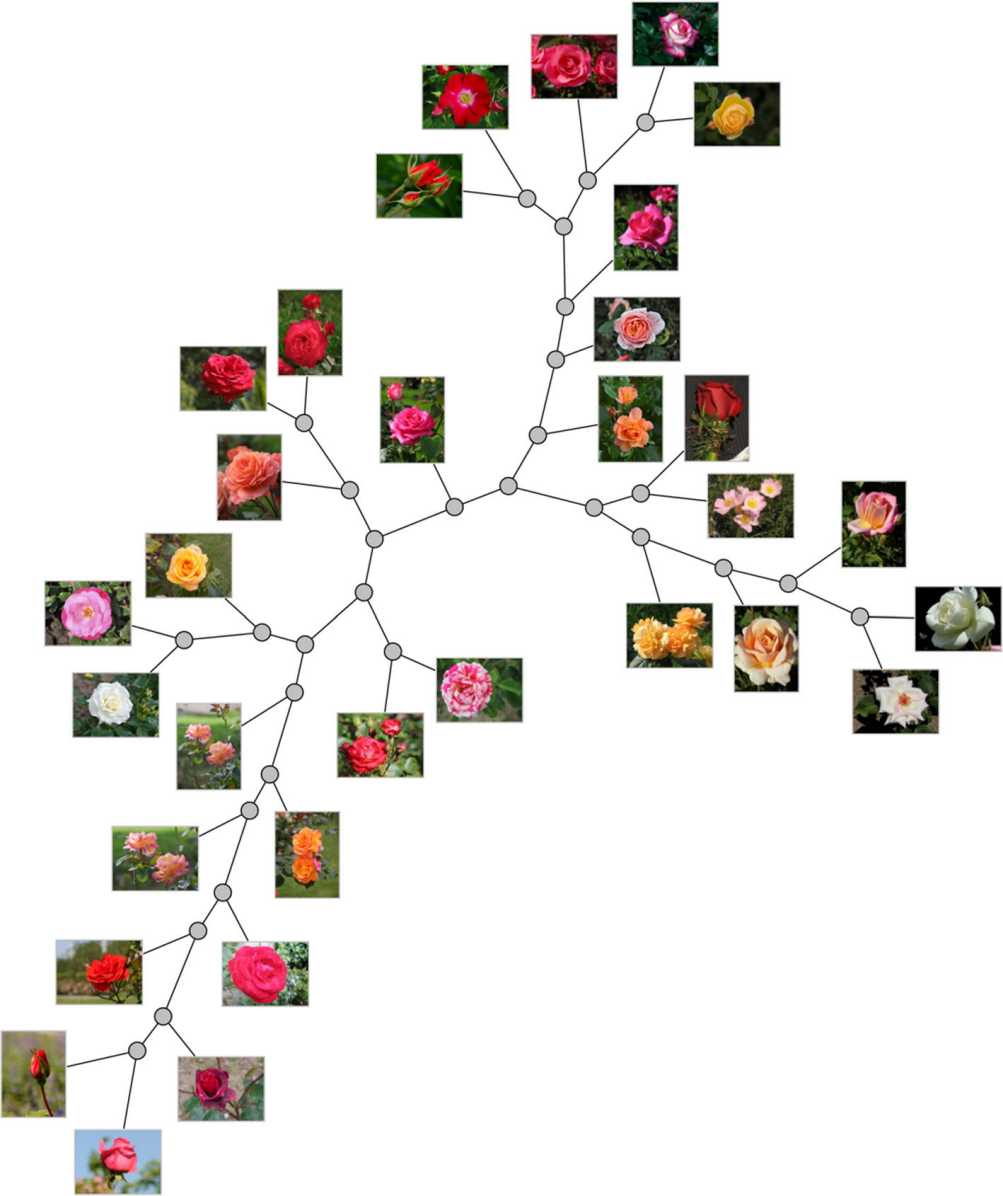
NJ tree of images. A visual Neighbor-Joining phylogeny for 32 images from Wikipedia Commons (https://commons.wikimedia.org)

The usage of a phylogeny as a point placement technique was analyzed elsewhere [2]. Figure 10 shows an example of the technique for a set of images. Interesting features of a visual phylogeny include the fact that the tree organizes data into branches of similarity that are amenable to exploration and provide a clearer separation among data items, both in small and large levels of zoom.

A disadvantage of visual phylogenies, when compared for instance to projections, is the occupation of visual space. A phylogenetic tree for *n* data items will have *n*−2 hypothetical nodes that represent hypothetical ancestors, but for text, images and other types of non-biological data, the notions of evolution and ancestor are not well defined unless a history of edition operations exists and is known. This is a consequence of the fact that measures of similarity among text, images and other non-biological data are not formulated to capture the notion of evolution, as measures of similarity for molecular sequences often do. Moreover, measuring similarity among data items is a hard problem by itself.

Nevertheless, we rely on the existing similarity measures for building phylogenies of non-biological data because such trees render a good layout for data exploration. Live Neighbor-Joining may be an interesting alternative to Neighbor-Joining in the construction of such visual maps because different relations of data may be revealed and also because a more compact layout may result, as the number of hypothetical nodes is potentially smaller.

Regarding the occupation of visual space, LNJ may be tuned to produce a more compact layout if we add a threshold for the comparison at Line 5 of the algorithm, turning the test into *S*_*ij*_<*αT*_*uvw*_, for a real *α*>0. Having *α* smaller than 1 will favor positioning data as internal nodes, and larger values of *α* will force LNJ to behave as NJ. Of course the difference among the distances in the tree and the distances in the input matrix will worsen with the reduction of *α*, but a useful balance may be reached in practice, and the introduction of *α* widens the applicability of LNJ.

**Fig. 11:**
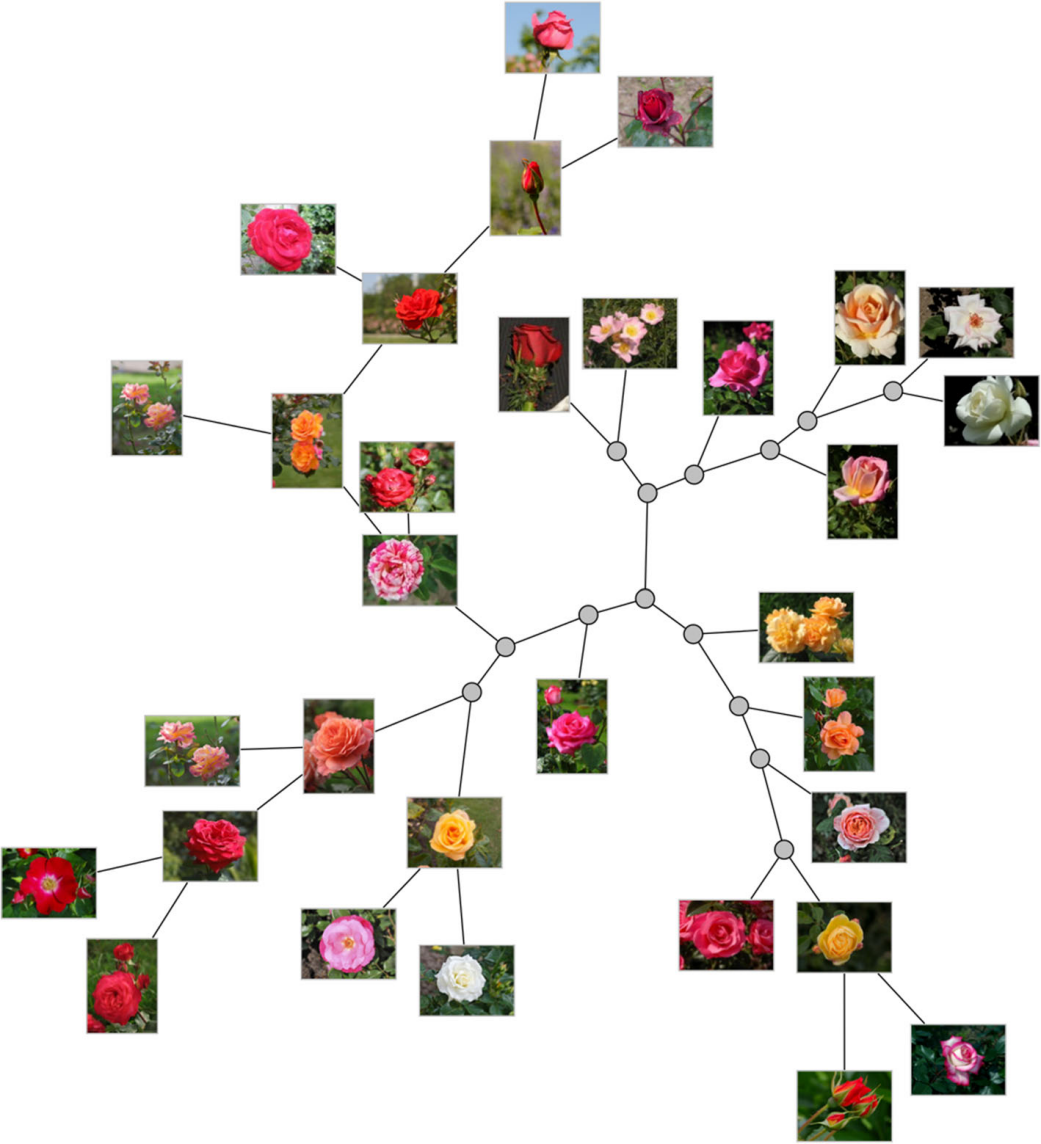
LNJ tree of images. A visual Live Neighbor-Joining phylogeny (*α*=0.9) for 32 images from Wikipedia Commons (https://commons.wikimedia.org), the same images of Fig. 10

**Fig. 12:**
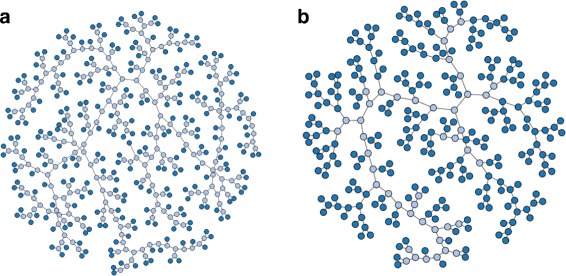
Visual phylogenies for 256 books. **a** NJ phylogeny with 510 nodes and 254 hypothetical ancestors. **b** LNJ phylogeny with 300 nodes and 105 live ancestors

The tree in Fig. 10 was built by Neighbor-Joining on pairwise distances evaluated by structural similarity [34] among 32 flower images from Wikipedia Commons (https://commons.wikimedia.org) trimmed and resized to 939×704 pixels. Figure 11 shows the Live Neighbor-Joining tree setting *α*=0.9, which has fewer internal nodes and preserves much of the local relations in the NJ tree. To further illustrate the space usage issue, Fig. 12a shows an NJ phylogeny for 256 free books from the Gutenberg project (http://www.gutenberg.org) with 510 nodes, and Fig. 12b shows an LNJ phylogeny for the same data with 300 nodes and 105 live ancestors. The books in ASCII format where processed for removal of Gutenberg Project’s preamble and license, and then the Normalized Compression Distance [35] for each pair of books was evaluated using bzip2. The nodes in the trees were positioned by a force-directed algorithm implemented in D3.js. Even more examples may be found in [2, 19].

Experience tells us that a data map with more than a thousand points just seems to be too much to explore at once, and that for even larger datasets a mutiscale approach combined with summarization techniques is imperative. Table 1 shows that Live Neighbor-Joining will be practical for the construction of layouts for a few hundred data items, and also suggests that a multiscale visualization that partitions the dataset may still use Live Neighbor-Joining to construct layouts at the finer levels of a visualization scheme.

## Conclusions

In this work we presented a new heuristic for the Distance-Based Live Phylogeny Problem. We first described the well-known Neighbor-Joining method that joins, at each step, a pair of taxa that gives the smallest sum of branch lengths. Such pair is joined into a new hypothetical internal node. Then we presented Live Neighbor-Joining, that extends the rationale of Neighbor-Joining by introducing the case where the creation of a live internal node results in a smaller sum of branch lengths. Thus, at each step of Live Neighbor-Joining, two options may apply: one as in Neighbor-Joining and another admitting a live internal node.

We applied Live Neighbor-Joining on three datasets of RNA virus genomes: Zika, Chikungunya and Ebola. In all cases, Live Neighbor-Joining presents alternative hypothesis for the relationship of the virus strains, providing researchers with a good environment for new investigations on the spreading of outbreaks.

Our experiments have focused on collections of viral genomes, which evolve quickly and may coexist in a real population. We also have presented experiments involving a set of bacteria. As pointed out in [2, 19], populations of non-biological data may also be analyzed through phylogenies. In particular, for collections of documents, like text processing files, web pages and images that may be subject to edition, the co-existence of different versions is also a fact. Such applications may also resort to Live Neighbor-Joining for alternative views on such data.

Live Neighbor-Joining is expensive, perhaps at the edge of practical applicability. In its favor we can point out that for up to a few hundreds taxa the running time is small, which fits many biological and non-biological datasets. Moreover, faster extensions that were already proposed for Neighbor-Joining may be applied to Live Neighbor-Joining, with pros and cons that must be addressed in future research.
